# Freshness Identification of Oysters Based on Colorimetric Sensor Array Combined with Image Processing and Visible Near-Infrared Spectroscopy

**DOI:** 10.3390/s22020683

**Published:** 2022-01-17

**Authors:** Binbin Guan, Wencui Kang, Hao Jiang, Mi Zhou, Hao Lin

**Affiliations:** 1 Nantong Food and Drug Supervision and Inspection Center, Nantong 226400, China; guanbinbinde@126.com (B.G.); ntsyj_zm@126.com (M.Z.); 2 School of Food and Biological Engineering, Jiangsu University, Zhenjiang 212013, China; wckang1993@163.com (W.K.); jh113997@163.com (H.J.)

**Keywords:** oysters, storage time, colorimetric sensor array, visible near-infrared spectroscopy, variable screening

## Abstract

Volatile organic compounds (VOCs) could be used as an indicator of the freshness of oysters. However, traditional characterization methods for VOCs have some disadvantages, such as having a high instrument cost, cumbersome pretreatment, and being time consuming. In this work, a fast and non-destructive method based on colorimetric sensor array (CSA) and visible near-infrared spectroscopy (VNIRS) was established to identify the freshness of oysters. Firstly, four color-sensitive dyes, which were sensitive to VOCs of oysters, were selected, and they were printed on a silica gel plate to obtain a CSA. Secondly, a charge coupled device (CCD) camera was used to obtain the “before” and “after” image of CSA. Thirdly, VNIS system obtained the reflected spectrum data of the CSA, which can not only obtain the color change information before and after the reaction of the CSA with the VOCs of oysters, but also reflect the changes in the internal structure of color-sensitive materials after the reaction of oysters’ VOCs. The pattern recognition results of VNIS data showed that the fresh oysters and stale oysters could be separated directly from the principal component analysis (PCA) score plot, and linear discriminant analysis (LDA) model based on variables selection methods could obtain a good performance for the freshness detection of oysters, and the recognition rate of the calibration set was 100%, while the recognition rate of the prediction set was 97.22%. The result demonstrated that the CSA, combined with VNIRS, showed great potential for VOCS measurement, and this research result provided a fast and nondestructive identification method for the freshness identification of oysters.

## 1. Introduction

Oysters are a kind of seafood with a fatty and tender texture, and are the most popular seafood in the world [1]. They contain rich nutrients, such as protein, calcium, iron, and zinc, and are known as “sea milk”. In addition, the large amount of glycogen contained in oysters helps digestion and absorption for the elderly and children, and these ingredients also have a variety of physiological activities, such as cancer prevention [2]. However, it is precisely because of their high water content, fragile muscle tissue, and active endogenous proteases that the oysters are easily contaminated by infection and may undergo subsequent decomposition mediated by bacteria and fungi during transportation and storage [3], leading to spoilage which affects the food quality and safety of oysters. Therefore, it is necessary to detect the quality of oysters during transportation, storage and processing. Quality characteristics of oysters mainly include texture, color, tenderness, pH value and freshness, among which freshness is the most important reference index for evaluating the quality and safety of seafood [3,4,5]. The traditional detection methods for the shelf life or freshness of oysters mainly include sensory, chemical, physical and microbial population evaluation [6,7,8,9]. However, sensory evaluation requires professional training and is easily influenced by subjective factors. Other methods, including chemical techniques and microbiological measurements, are time consuming, destructive, and laborious. Therefore, it is necessary to develop a fast and non-destructive method for oyster freshness evaluation.

During the storage process, changes in the nutritional content of oysters is closely related to the volatile organic compounds (VOCs). For instance, the intensity of VOCs (lilac aldehyde, pentanal and 2,6-nonadienal) increased significantly during storage [10]. In addition, flavor is among the main indicators for sensor analysis and quality estimation [11]. Hence, VOCs can be used as an indicator of the freshness of oysters. The traditional VOCs detection method is mainly gas chromatography-mass spectrometer (GC-MS). However, this method has some disadvantages, such as the high instrument cost, being destructive to samples, cumbersome pretreatment, and being time consuming. Compared with GC-MS, the electronic nose mainly uses gas sensors to capture the VOCs of the sample and then converts it into electrical signals. Although, compared with GC-MS, it has the advantages of being time saving, low cost, simple to operate, and non-destructive, the electronic nose was affected by the humidity of the working environment which can lead to signal drift [12]. The colorimetric sensor array was first put forward by Kenneth Suslick [13] and is a new method of characterizing VOCs that has emerged in recent years. Colorimetric sensor array was composed by chemical dyes that are sensitive to the specific VOCs (which can change colors after exposure to specific VOCs). The color difference value of the colorimetric sensor array before and after exposure to specific VOCs can be obtained by a charge coupled device (CCD) camera for qualitative and quantitative analysis, which could express smell information through color changes. Colorimetric sensors have been widely applied for aroma quality evaluation of pork, fish, tea, wine, vinegar and other kinds of food [14,15,16,17,18,19]. However, the change information that is obtained based on the colorimetric sensor array is mainly characterized by extracting the RGB difference images before and after the reaction; that is, the color change of each color-sensitive material is only characterized by the three components of R, G, and B. Therefore, less effective amounts of information may limit the correctness of judging the storage time of oysters to a certain extent. Near-infrared spectroscopy is a physical technique with the advantages of simple operation and rapid detection. However, it cannot directly detect gaseous compounds. What is more, due to the high water content of raw oyster samples, the freshness of raw oysters detected by near-infrared spectroscopy technology directly alone would likely be affected by humidity. At present, our research group has carried out a series of experiments on the combination of near-infrared spectrometers and colorimetric sensor array [20]. Visible near-infrared spectroscopy combines the colorimetric sensor array method with near-infrared technology. On one hand, it avoids the influence of humidity on the near-infrared technology and has the advantage of high accuracy; on the other hand, it not only reflects the color change, but also reflects changes in the internal structure of color-sensitive materials.

In this study, the colorimetric sensor array, combined with image processing and visible near-infrared spectroscopy methods were developed to discriminate the freshness of oysters. The performance of the colorimetric sensor array in oyster freshness identification was analyzed and compared with the visible near-infrared spectroscopy method. On this basis, linear discriminant analysis (LDA) and the K-nearest neighbors (KNN) model for oyster storage time, based on the visible near-infrared spectroscopy method, was further optimized through the selection of different variable screening algorithms. 

## 2. Materials and Methods

### 2.1. Materials

Fresh and live oysters of the same batch were purchased from Zhenjiang Yonghui Supermarket. Raw oysters of similar size were selected, with each oyster weighing about 100–120 g/piece, and all raw oysters had not been processed for moisture absorption. Afterwards, they were placed in a refrigerator at 4 °C for 0 day, 2 days, 4 days, 6 days, 8 days, and 10 days, and were accordingly divided into six groups. There were 30 samples in each group, for a total of 180 samples. These 180 oyster samples were divided into five parts randomly in later pattern recognition, three of which were used as training set samples (108 samples), and two of which were used as prediction set samples (72 samples). In this study, the raw oysters being purchased were all fresh and live, and the freshness was qualitatively determined based on the storage days of oysters stored at in the refrigerator at 4 °C. 

### 2.2. Colorimetric Sensor Array Image Data Acquisition

**Figure 1:**
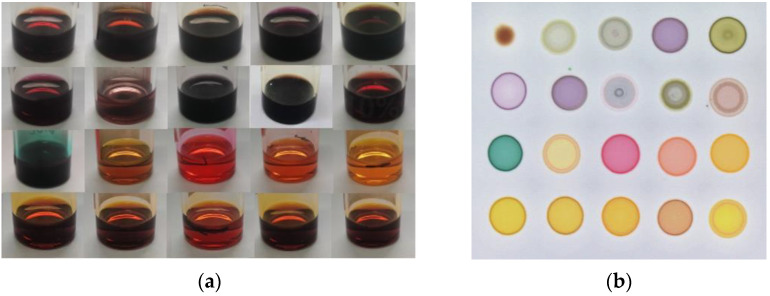
20 color-sensitive materials were dissolved in DMF (**a**) and a 4 × 5 colorimetric sensor array (**b**).

In this study, color-sensitive materials were synthesized according to the classic Lindsey methodology in the laboratory [20,21]. Twenty color-sensitive materials were dissolved in N,N-Dimethylformamide (DMF) with a concentration of 2.0 mg/mL, as shown in Figure 1a, and then poured into a silica gel plate (Merck, Germany) through a capillary tube (0.5 mm × 100 mm), structing a 4 × 5 sensor array, as shown in Figure 1b. 

**Figure 2:**
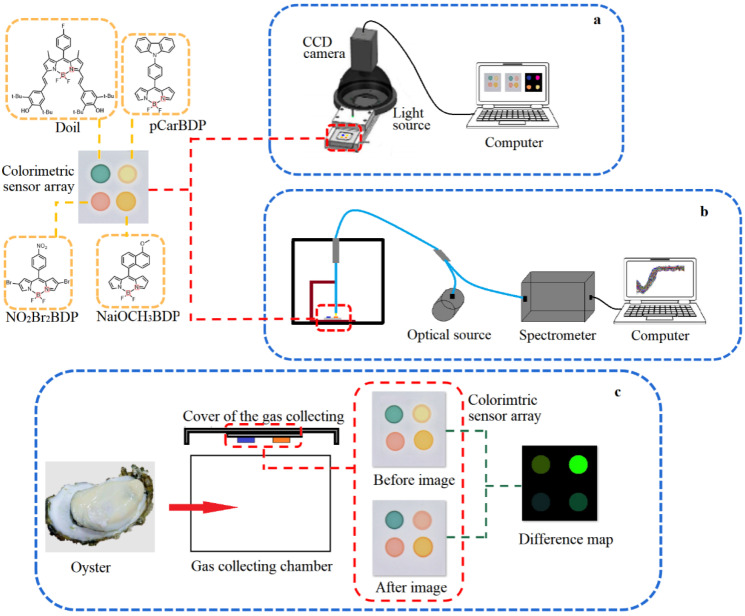
The schematic diagram of colorimetric sensor array system (**a**), visible near-infrared spectroscopy system (**b**) and difference map acquisition of oysters (**c**).

The schematic diagram of the colorimetric sensor array is shown in Figure 2a. The CCD camera recorded the “before image” of the colorimetric sensor array before it was exposed to the VOCs of the oysters. After exposure to the VOCs for 10 min at the temperature of 20 °C, the CCD camera recorded the “after image”. A special software program performed analysis of the signal from the camera. Every color-sensitive material is expressed by the red (R), green (G), and blue (B) value. Table 1 shows the “mean and standard” difference value of 20 color-sensitive materials after exposure to the oysters’ VOCs. Considering the large response difference and small standard deviation, (4,4′-difluoro-8-(methyl 4-benzoate)-1,7-dimethyl-2,6-diethyl-3,5,-distyryl-(3,5-di-tert-butyl-4-hydroxyphenyl)-4-bora-3a,4a-diaza-s-indacene (Doil), 8-(4-Carbazolephenyl)-4,4-difluoroboron dipyrromethane (pCarBDP), 8-(4-Nitrophenyl)-4,4-difluoro-2,6-dibromoborin dipyrrole (NO_2_Br_2_BDP), and 8-(6-methoxy-2-naphthyl)-4,4-difluoroboron dipyrromethane (NaiOCH_3_BDP) were selected as the color-sensitive materials. 

As seen from Figure 2c, a 2 × 2 sensor array was constructed to determine the VOCs of oysters with different storage times (stored at 4 °C for 0 day, 2 days, 4 days, 6 days, 8 days, and 10 days, with 30 samples in each group for a total of 180 samples). After being recorded as the “before image”, the colorimetric sensor array was fixed on the cover of the gas collecting chamber, and the oyster samples were placed in the gas collecting chamber. The cover was quickly applied to make sure that the colorimetric sensor array was fully exposed to the VOCs. The CCD camera recorded the “after image” 10 min later. After the reaction, the average gray values of components R, G, and B in the region of interest (ROI) were obtained and subtracted to get the feature difference before and after the reaction, which could standardize all of the response differences in the same measurement and avoid the matrix effect of the colorimetric sensor array sensitivity before the reaction. After processing with a specific image processing software, all data (180 samples) for the vector (RGB color space model (R, G, B), HSV color space model (hue, saturation, value), laboratory color space model (L, a, b), and eigenvalues ($\sqrt{R^{2}+G^{2}+B^{2}}$) for each of the four dyes in the array, a total of 40 variables) were used in statistical and subsequent pattern recognition. 

### 2.3. Visible Near-Infrared Spectroscopy Data Acquisition

The schematic diagram of a visible near-infrared spectroscopy system was shown as Figure 2b. After the VOCs of the oysters fully react with the colorimetric sensor array for 10 min, the colorimetric sensor array was taken out and placed in the visible near-infrared spectroscopy acquisition device, and the reflected spectrum data of the colorimetric sensor array after reaction was collected.

The spectrum acquisition parameters are set as follows: the integration time is 100 ms, the smoothness is five, and the number of averages is 10 times. The spectral range was 899.20~1724.71 nm, wavenumber interval was 1.66 nm with 512 variables. Each color-sensitive material can obtain three pieces of spectral data. Therefore, a total of 540 spectra data were collected and each spectrum had a total of 2048 variables. The temperature in the laboratory was kept at around 20 °C, and the humidity was maintained at a stable level.

### 2.4. Variable Screening of Visible Near-Infrared Spectroscopy Data

In order to eliminate or weaken the influence of the difference of the samples during the sampling process, as well as the scattering and optical path change during the sampling process, firstly the spectrum was preprocessed through standard normal variate transformation (SNV) [22,23]. In addition, due to the high-dimensional and high correlation characteristics of the near-infrared spectroscopy data, the obtained near-infrared spectroscopy data were too large, and the hydrogen-containing groups had different levels of frequency doubling and combined frequency absorption in the near-infrared spectral region, resulting in a large amount of overlapping redundant information for the severe overlap of absorption peaks in the near-infrared spectroscopy [24]. In order to reduce the blindness in the selection of spectral variables and narrow the search range, synergy interval partial least square (siPLS) was used to screen the characteristic bands. 

### 2.5. Multivariate Statistical Analysis

Multivariate analysis methods play a key role in characterizing the VOCs of oyster samples with different storage times based on the colorimetric sensor array and visible near-infrared spectroscopy. All algorithms were implemented in MATLAB R2016b (Mathworks, Natick, MA, USA) under Windows 10.

## 3. Results

### 3.1. Image Characterization of Oysters Stored for Different Times by Colorimetric Sensor Array

**Figure 3:**
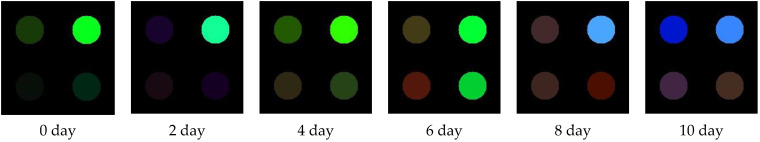
Color difference diagram of colorimetric sensor array before and after the reaction of the oysters’ VOCs with different storage time.

Average color change profiles were obtained from oyster samples with different storage times. Figure 3 shows the difference maps of the VOCs of oyster samples with a storage time of 0, 2, 4, 6, 8 and 10 days of being exposed to the colorimetric sensor array. As shown in Figure 3, the colorimetric sensor array has its specific colorific fingerprint of the VOCs from the oyster samples with different storage times, which indicates that the oyster samples at different storage periods have their specific VOCs.

### 3.2. Results of Colorimetric Sensor Array Combined with Image Processing

**Figure 4:**
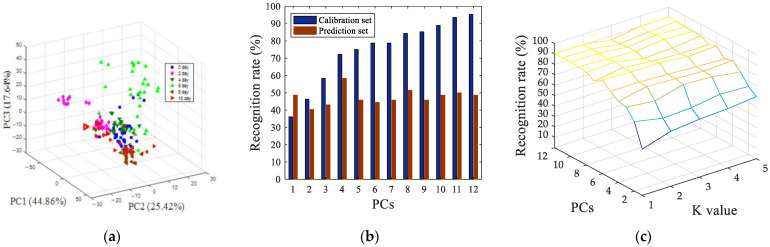
Pattern recognition results of PCA (**a**), LDA (**b**) and KNN (**c**), based on colorimetric sensor array.

The data from the colorimetric sensor variables contained overlapping information. In order to extract useful information from the original data, principal component analysis (PCA) was used to present the oyster storage trends in an intuitive way. Geometrical exploration, based on the PCA score plots, shows the cluster trend in 3-dimension (3D) space. Figure 4a shows a 3D space of all of the oysters samples with different storage times, represented by PC1, PC2 and PC3. The cumulative variance contribution rate of the first three principal components reached 87.92% (PC1 was 44.86%, PC2 was 25.42%, and PC3 was 17.64%). It can be seen from Figure 4a that the oyster samples of different storage days have a certain clustering trend in the figure, but it is not very obvious. This can be explained, on the one hand, as there being differences between the samples themselves, and, on the other hand, as the corruption itself being a continuity.

In order to further investigate the freshness characterization using the colorimetric sensor array, the linear discriminant analysis (LDA) model and K-nearest neighbors (KNN) were used to discriminate the storage times of the oyster samples. PCA scores were input into the LDA and KNN algorithm as latent variables. The input of each model was the score of each principal component, and the output was the category corresponding to the oysters of different storage days, and then the LDA and KNN pattern recognition were performed. Prediction results for identification of oyster storage times, based on LDA, were shown in Figure 4b, and the result indicates that when the number of principal components was four, only 58.33% of the prediction samples were correctly identified. Prediction results for the identification of oyster storage times, based on KNN, was shown in Figure 4c, when the number of principal components was nine and K value was one, the best recognition rate of prediction set was 90.28%, at this time, and the recognition rate of the training set was 95.37%. The recognition result of the KNN model is much better than LDA, on account of the non-linear changing of the oysters’ VOCs during storage. The result of the nonlinear pattern recognition model KNN was better than that of the linear pattern recognition model LDA.

### 3.3. Results of Colorimetric Sensor Array Combined with Visible Near-Infrared Spectroscopy

**Figure 5:**
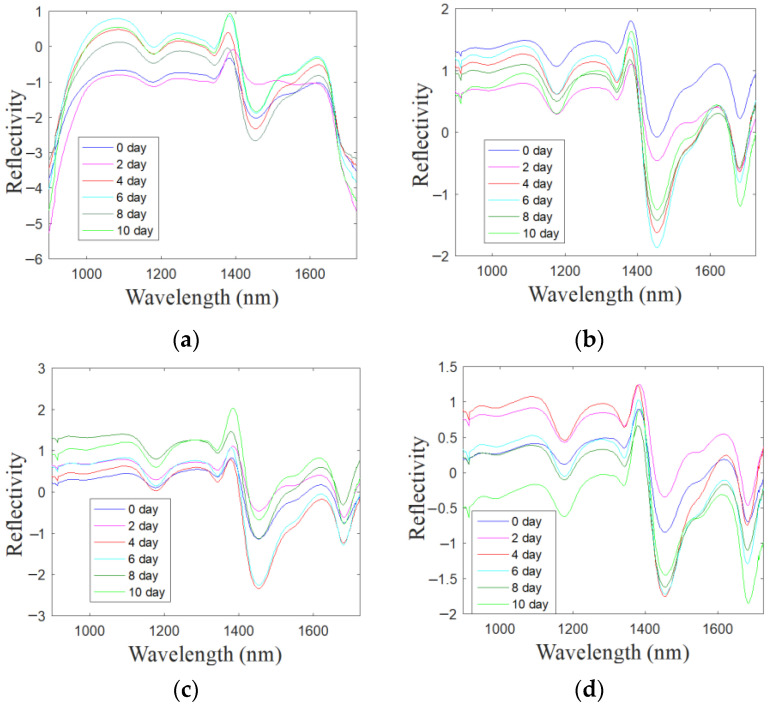
Average spectrum of Doil (**a**), pCarBDP (**b**), NO2Br2BDP (**c**) and NaiOCH3BDP (**d**) after SNV pretreatment.

Color-sensitive materials were reacted with the VOCs of 180 oyster samples with different storage times, and the spectral data of four kinds of color-sensitive materials were then extracted, and a total of 720 spectral curves were obtained. Through calculation, the average spectral curves of the oysters collected by the four color-sensitive materials (Doil, pCarBDP, NO_2_Br_2_BDP and NaiOCH_3_BDP) during different storage periods were obtained. The result was shown in Figure 5. It can be found that after reacting with the oysters’ VOCs of different storage times, the spectrum obtained by each color-sensitive material is different, which indicates that the color reaction of the color-sensitive material will be different due to the difference in storage time. 

Given that each sensor has four dyes, there are a total of 2048 variables, which are too many. SiPLS is used to divide the spectral interval first, and the result shows that when the spectrum were divided into 16 sub-intervals, and one, two, three, and four sub-intervals were used to establish a joint interval, the principal component is nine, and the cross-validation root mean square error (RMSECV) value is smallest, at 0.2711. At this time, the total number of variables has been reduced from 2048 to 512.

Although siPLS has reduced the data dimension, 512 variables still have a computational burden on the establishment of the oyster freshness prediction model. Therefore, three different variable screening algorithms were used to select the characteristic wavelengths. Competitive adaptive reweighted sampling (CARS) uses adaptive heavy weighted sampling (ARS) to select the wavelength points with large absolute value of the regression coefficient in the PLS model, remove the wavelengths with small weight, and use interactive verification to select the lowest subset of the root mean square error of prediction (RMSECV) [25,26], which can effectively provide the optimal combination of variables. As an intelligent optimization algorithm, the genetic algorithm (GA) uses the global search function and continuously performs genetic iterations to achieve the best results [27]. The ant colony optimization (ACO) method is mainly based on the way that ants search for food, through global cooperation between all individuals in the group, and constantly exchange path information via pheromones to find the optimal solution [28]. As such, the variables selected by the three variable selection algorithms were qualitatively judged by the KNN and LDA pattern recognition models.

Table 2 shows the identification result of qualitative analysis. It was indicated that the LDA model and KNN model can be used for the oyster freshness characteristic recognition. Overall, the recognition effect of the LDA model is better than the KNN model. Compared to CARS, the LDA model after ACO and GA variable screening algorithms obtains the better results, as the recognition rate of the training set were 100%, and the recognition rate of the prediction set were 97.22%. In addition, the number of principal component factors of GA was less; when the principal component factor was only nine, the LDA model after GA variable screening algorithm obtained the best classification result.

**Figure 6:**
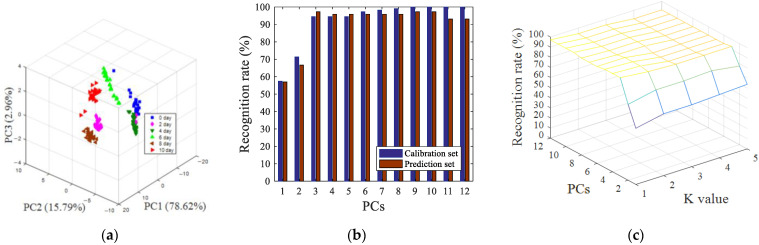
Pattern recognition results of PCA (**a**), LDA (**b**) and KNN (**c**) based on visible near-infrared spectroscopy data after SNV preprocessing, siPLS interval screening, and GA variable screening.

After SNV preprocessing, siPLS interval screening, and GA variable screening, the pattern recognition results of visual near-infrared spectroscopy data were shown in Figure 6. Figure 6a shows a 3D space of all of the oyster samples with different storage time, the cumulative variance contribution rate of the first three principal components reached 97.37% (PC1 was 78.62%, PC2 was 15.79%, and PC3 was 2.96%). It can be seen from Figure 6a that, compared with the parameters of image processing, the clusters of samples between different categories were more clustered. The fresh oysters (0 days) could be directly distinguished from samples which were stored at 4 °C for more than four days. The LDA model and KNN model were also used to predict the storage time of oyster samples, as shown in Figure 6b, when the number of principal component factors was nine, 100% of the calibration set samples were classified correctly, and 97.22% of the prediction set samples were classified correctly, as indicated in Figure 6c. When the number of principal components was 11 and the K value was 1, the best recognition rate of the prediction set was 97.22%, and the recognition rate of the calibration set was 99.07%. 

## 4. Conclusions

In this work, the colorimetric sensor array and visible near-infrared spectroscopy system were developed for oyster storage time identification. The characterization of the VOCs in the storage process of oysters by color-sensitive sensors combined with visual near-infrared spectroscopy could identify the freshness of oysters quickly and intuitively. The colorimetric sensor array was used firstly to collect the VOCs. However, when the color-sensitive sensor was exposed to the VOCs of oysters, which not only reflects as the color change but also reflects changes in the internal structure of color-sensitive materials, the near-infrared spectroscopy was used to analyze the VOCs information related to oyster freshness. Furthermore, if the near-infrared spectroscopy was used directly on the oyster samples for detection, the high water content of raw oyster samples may impact on the performance. As such, in this study, the visible near-infrared spectroscopy system was used to obtain the reflected spectrum data of the colorimetric sensor array. SNV was applied to preprocess the spectrum to eliminate the effects of solid particles, light intensity variation, and surface scattering on the spectra, and siPLS was applied to reduce the data dimension. The variable screening algorithms (CARS, ACO and GA) were used to select the effective wavelengths for oyster freshness detection. The results show that the GA variable screening algorithm obtains the best classification result. Compared with a separate colorimetric sensor array image data model, the visible near-infrared spectroscopy method exhibited promising performance in terms of the identification of oyster storage times. On one hand, the NIR provides more information based on the large amount variables, and in combination with the optimization algorithm, could be also helpful in achieving better performance. On the other hand, when the color-sensitive sensor is exposed to the VOCs of oysters, which not only reflects the color change but also reflects changes in the internal structure of color-sensitive materials, the NIR could collect the information of the color-sensitive material in the invisible wavelength range. Therefore, the recognition effect was better after adding the NIR. The fresh and stale oyster samples could be separated directly from the PCA score plot. When the number of principal component factors was 9, 100% of the calibration set samples were classified correctly, and 97.22% of the prediction set samples were classified correctly in the LDA model.

Furthermore, the colorimetric sensor array composed by the color-sensitive material modified with porous silica nanosphere is under study.

## Figures and Tables

**Table 1:** The difference value of 20 color-sensitive materials after exposed to the oysters’ VOCs.

Color-Sensitive Materials	R Component ^1^	G Component ^1^	B Component ^1^
2,3,7,8,12,13,17,18-Octaethyl-21H,23H-porphine manganese(III) chloride	3.51 ± 2.31	13.44 ± 1.66	2.28 ± 1.32
5,10,15,20-Tetrakis(4-methoxyphenyl)-21H,23H-porphine iron(III) chloride	3.69 ± 3.99	3.99 ± 1.46	3.12 ± 1.28
5,10,15,20-Tetraphenyl-21H,23H-porphine iron(III) chloride	4.17 ± 4.45	2.62 ± 0.91	2.75 ± 1.38
5,10,15,20-tetra(4-methoxyphenyl)Porphyrin Fe(II) complex	4.29 ± 0.81	3.41 ± 0.98	3.43 ± 1.32
5,10,15,20-Tetrakis(4-sulfonatophenyl)-21H,23H-porphine manganese(III) chloride	3.73 ± 0.54	4.57 ± 2.12	3.04 ± 1.16
5,10,15,20-Tetraphenyl-21H,23H-porphine nickel(II)	3.35 ± 2.11	4.63 ± 2.47	4.45 ± 1.02
5,10,15,20-Tetraphenyl-21H,23H-porphine palladium(II)	2.92 ± 0.74	4.96 ± 3.52	23.50 ± 2.94
5,10,15,20-Tetraphenyl-21H,23H-porphine palladium(II)	2.44 ± 0.95	2.16 ± 0.90	3.36 ± 2.93
meso-tetra(4-sulfonic) porphine tetrasodium dodecahydrate	2.06 ± 0.74	0.78 ± 0.47	4.67 ± 0.82
5,10,15,20-Tetraphenyl-21H,23H-porphine cobalt(II)	0.87 ± 0.53	1.37 ± 0.86	11.14 ± 1.07
4,4′-difluoro-8-(methyl 4-benzoate)-1,7-dimethyl-2,6-diethyl-3,5,-distyryl-(3,5-di-tert-butyl-4-hydroxyphenyl)-4-bora-3a,4a-diaza-s-indacene	1.52 ± 0.43	4.10 ± 1.18	29.13 ± 2.24
8-(4-Carbazolephenyl)-4,4-difluoroboron dipyrromethane	13.49 ± 1.85	7.15 ± 0.97	4.43 ± 1.05
8-(4-Nitrophenyl)-4,4-difluoro-6-bromoborin dipyrromethane	2.66 ± 1.21	3.24 ± 1.73	10.89 ± 1.45
8-(4-Nitrophenyl)-4,4-difluoro-2,6-dibromoborin dipyrrole	3.44 ± 0.99	3.58 ± 0.83	30.66 ± 3.20
8-(6-methoxy-2-naphthyl)-4,4-difluoroboron dipyrromethane	2.10 ± 1.24	15.06 ± 3.09	4.26 ± 1.45
Bis (8-phenyldipyrromethane) nickel(II)	3.83 ± 0.85	1.15 ± 0.80	5.02 ± 2.19
Bis [8-(4-formylformylphenyl) dipyrromethane] nickel (II)	2.70 ± 0.62	7.22 ± 1.51	1.53 ± 2.23
Bis [8-(6-methoxy-2-naphthyl)dipyrromethane] nickel(II)	3.43 ± 0.85	2.72 ± 0.42	2.65 ± 1.20
Di [8-(4-carbazolephenyl) dipyrromethane] copper(II)	0.97 ± 0.54	2.34 ± 1.14	10.57 ± 4.75
Bis [8-(4-carbazolylphenyl) dipyrromethane] zinc(II)	1.33 ± 0.49	3.09 ± 0.95	6.43 ± 2.32

^1^ Mean ± standard.

**Table 2:** LDA and KNN classification results of three variable screening algorithms.

Variable Screening Algorithms	LDA			KNN			
	PCs	Rc	Rp	PCs	K Value	Rc	Rp
ACO	11	100%	97.22%	6	1	99.07%	94.44%
CARS	3	90.74%	94.44%	7	1	96.30%	93.06%
GA	9	100%	97.22%	11	1	99.07%	97.22%

## Data Availability

Not applicable.
